# Preventive Effects of *Rhodiola rosea* L. on Bleomycin-Induced Pulmonary Fibrosis in Rats

**DOI:** 10.3390/ijms17060879

**Published:** 2016-06-03

**Authors:** Ke Zhang, Xiao-Ping Si, Jian Huang, Jian Han, Xu Liang, Xiao-Bo Xu, Yi-Ting Wang, Guo-Yu Li, Hang-Yu Wang, Jin-Hui Wang

**Affiliations:** 1 School of Traditional Chinese Materia Medica, Shenyang Pharmaceutical University, Shenyang 110016, China; xjzk1984@163.com (K.Z.); 13998302950@163.com (J.H.); xxb517234556@sina.com (X.-B.X.); 2 Shihezi Institute for Drug Control, Shihezi 832002, China; shz_yjs@sina.com; 3 College of Pharmacy, Shihezi University, Shihezi 832002, China; lix8182@163.com (X.L.); christina.wyt@foxmail.com (Y.-T.W.); liguoyulisa@163.com (G.-Y.L.); why_pha@shzu.edu.cn (H.-Y.W.)

**Keywords:** *Rhodiola rosea* L., pulmonary fibrosis, bleomycin, TGF-β1, MMP-9

## Abstract

*Rhodiola rosea* L. (RRL) possesses a wide range of pharmacological properties, including lung-protective activity, and has been utilized in folk medicine for several 100 years. However, the lung-protective mechanism remains unclear. This study investigated the possible lung-protective activity mechanism of RRL in a pulmonary fibrosis (PF) rat model. Lung fibrotic injury was induced in Sprague–Dawley rats by single intratracheal instillation of saline containing bleomycin (BLM; 5 mg/kg). The rats were administered 125, 250, or 500 mg/kg of a 95% ethanol extract of RRL for 28 days. The animals were killed to detect changes in body weight, serum levels of glutathione (GSH) and total superoxide dismutase (T-SOD), as well as lung tissue hydroxyproline (HYP) content. Tumor necrosis factor-α (TNF-α), transforming growth factor-β1 (TGF-β1), and interleukin 6 (IL-6) levels were measured in bronchoalveolar lavage fluid (BALF) by enzyme-linked immunosorbent assay. Hematoxylin and eosin, Masson’s trichrome, and immunohistochemical staining were performed to observe the histopathological changes in lung tissues. Additionally, target-related proteins were measured by Western blotting. RRL alleviated the loss of body weight induced by instilling BLM in PF rats, particularly at the 500 mg/kg per day dose. RRL reduced HYP (*p* < 0.01) and increased GSH and T-SOD contents. BALF levels of TNF-α, TGF-β1, and IL-6 decreased significantly in the RRL-treated groups. Expression levels of matrix metalloproteinase-9 (MMP-9) and α-smooth muscle actin decreased significantly in a dose-dependent manner in response to RRL. Moreover, the levels of TGF-β1 and tissue inhibitor of metalloproteinase-1 in lung tissues also decreased in the RRL-treated groups. RRL alleviated BLM-induced PF in rats. Our results reveal that the protective effects of RRL against fibrotic lung injury in rats are correlated with its anti-inflammatory, antioxidative, and anti-fibrotic properties. MMP-9 may play important roles in BLM-induced PF.

## 1. Introduction

Pulmonary fibrosis (PF) is a progressive interstitial lung disease characterized by excessive proliferation of fibroblasts and deposition of collagens and other extracellular matrix (ECM) proteins [1]. PF is induced by a variety of etiological factors, including inflammation, the epithelial–mesenchymal transition (EMT), oxidative stress, and immune dysfunction, which result in alveolar epithelial cell injury, and fibroblast proliferation that consequently lead to abnormal deposition of the extracellular matrix (ECM) and tissue remodeling. A balanced turnover of the ECM through regulation of myofibroblast synthesis and degradation by matrix metalloproteinases (MMPs) is critical for proper formation. This process is featured by the presence of α-smooth muscle actin (α-SMA) and high synthetic rates of ECM components including collagens [2]. MMPs and tissue inhibitors of metalloproteinases (TIMPs) comprise an important enzyme system that regulates degradation of the cellular matrix. Shan-Zhong Tan *et al.* [3] suggested that MMPs and TIMPs are involved in degradation of the ECM and basement membrane during early PF (injury stage), whereas they play an important role regulating remodeling of lung tissue structure in the late stages of PF. Many factors change the expression of MMPs, including pro-inflammatory cytokines, such as tumor necrosis factor-α (TNF-α) and interleukin-6 (IL-6), which increase during the early phase of PF and are essential in the progression of early pulmonary inflammation to PF [4,5]. These changes have been associated with increased MMP levels [6] and induction of MMP production from various cell types, including alveolar epithelial cells, alveolar fibroblasts, and alveolar macrophages [7,8,9]. Some studies have also shown that other cytokines, such as transforming growth factor-β1 (TGF-β1), regulate MMP-9 and TIMP-1 expression, suggesting that these cytokines may play roles in pulmonary cells and promote PF through synergistic actions with TGF-β1. However, the PF mechanism is not completely understood, and novel therapeutic agents to treat PF are still needed.

*Rhodiola rosea* L. (RRL; Crassulaceae family), also known as golden root or rose root, has a long history as traditional Chinese medicine (TCM) with medicinal efficacy similar to that of ginseng and Many prickle Acathopanax roots. RRL is a perennial herbaceous plant widely distributed at high altitudes on rocks and on Arctic sea cliffs in Europe, Asia, and North America [10]. The dried roots and rhizomes of RRL are called Hong-Jing-Tian in Chinese [11] and are traditionally used as a tonic and adaptogen in TCM and as a hemostatic in Tibetan folk medicine. The pharmacological effects of RRL preparations are attributed to the phenylpropanoids, organic acids, and flavonoids extracted from the roots and rhizomes. RRL preparations exhibit adaptogenic effects, including neuroprotective, cardioprotective, anti-fatigue, anti-depressive, anxiolytic [12], nootropic, hepatoprotective, anti-allergic, anti-cancer, and life-span increasing effects, as well as central nervous system stimulating [13,14,15], anti-nociceptive, and anti-inflammatory activities [16,17]. These earlier studies mainly focused on the anti-inflammatory and anti-apoptotic/pro-apoptotic effects of salidroside in lung tissues and/or cells but not on its anti-fibrotic potential. One previous study revealed a novel anti-fibrotic effect of salidroside in the liver [18], which prompted us to investigate whether it is also effective in alleviating fibrotic lung injury. Thus, the aim of the present study was to investigate the anti-fibrotic effect and possible mechanism of an RRL extract on bleomycin (BLM)-induced PF in rats.

## 2. Results

### 2.1. RRL Mitigates Inflammation in Rats with BLM-Induced Pulmonary Fibrosis

**Figure 1:**
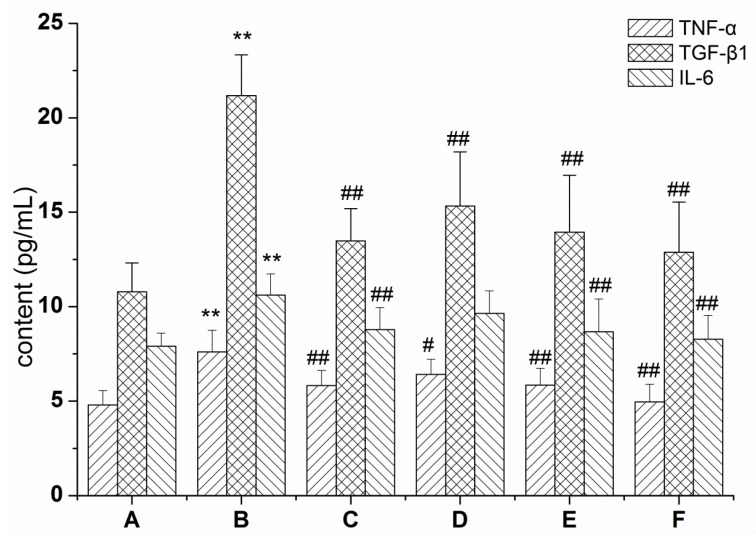
Effects of RRL (*Rhodiola rosea* L.) on the levels of transforming growth TNF-α (tumor necrosis factor-α), TGF-β1 (transforming growth factor-β1) and IL-6 (interleukin 6) in the BALF (bronchoalveolar lavage fluid) of BLM (bleomycin)-treated rats. (** *p* < 0.01 *vs.* the normal control group; ^#^ *p* < 0.05 *vs.* the BLM-treated group; ^##^ *p* < 0.01 *vs.* the BLM group). (A) normal group; (B) model group; (C) PAG (prednisone acetate) group; (D–F) are RRL group treated with 125, 250 and 500 mg/kg.

To determine the effect of RRL on the BLM-induced pulmonary inflammatory response in rats, levels of the inflammatory cytokines TGF-β1, TNF-α, and IL-6 were determined in BALF. As a result, TGF-β1, TNF-α, and IL-6 protein levels in BALF of the model group increased significantly after 28 days compared with those in the normal group (*p <* 0.01), whereas these levels in the treatment group were significantly lower than those in the model group after 28 days (*p <* 0.01) (Figure 1). These data show that the RRL treatment lowered levels of the TGF-β1, TNF-α, and IL-6 proteins in BALF, indicating that RRL has anti-inflammatory properties in rats with BLM-induced lung fibrosis.

### 2.2. RRL Alleviates Pathological Changes

**Figure 2:**
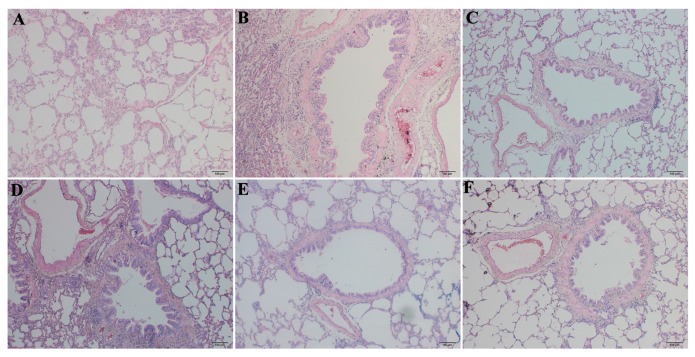
The H & E of the lung tissues in rats (Bar = 100 μm). (**A**) normal group; (**B**) model group; (**C**) PAG group; (**D**–**F**) are RRL groups treated with 125, 250 and 500 mg/kg, respectively.

H & E and Masson’s trichrome staining were applied to confirm establishment of the BLM-induced PF rat model. The H & E results after 28 days showed normal pulmonary alveoli in the normal group and no pathological changes, such as alveolitis or interstitial pulmonary fibrosis. Severe PF was observed in lungs from the BLM group (Figure 2), including marked thickening of alveolar septa, collapsed alveolar spaces, loss of alveolar structure, and over-proliferation of fibroblasts. The model group showed predominant infiltration of mononuclear cells rather than accumulation of neutrophils in the alveolar compartment. The 125 mg/kg RRL treatment slightly ameliorated the BLM-induced pathological changes in the lungs, whereas the 250 and 500 mg/kg RRL doses significantly alleviated BLM-induced PF.

**Figure 3:**
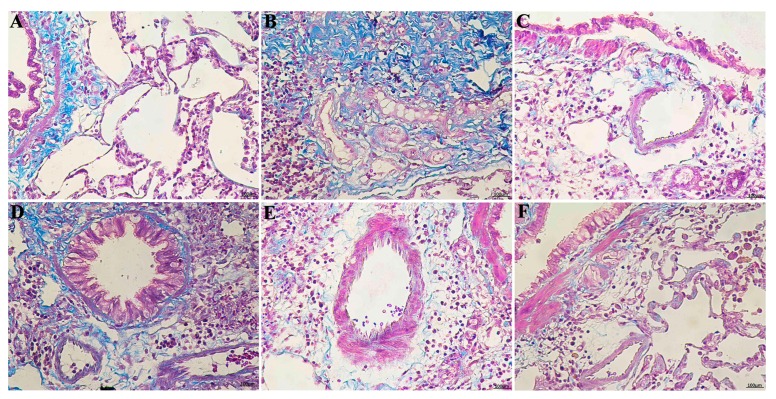
The Masson examinations of the lung tissues in rats (Bar = 100 μm). (**A**) normal group; (**B**) model group; (**C**) PAG group; (**D**–**F**) are RRL groups treated with 125, 250 and 500 mg/kg, respectively.

Masson’s trichrome staining of lung specimens demonstrated that BLM severely distorted lung structure and collagen fibers (blue) accumulated in the rat lungs. Figure 3B depicts the marked deposition of deep blue-stained mature collagen fibers surrounding the pulmonary vessels in the model group. Collagen deposition in the RRL group was significantly reduced compared with that in the model group. These results show that RRL alleviated collagen deposition and inflammation induced by BLM.

### 2.3. RRL Reduces the BLM-Induced Loss of Body Weight in Rats

As shown in Table 1, the body weights of the rats decreased significantly seven days after the BLM treatment commenced and decreased gradually thereafter (*n* = 10, *p <* 0.01). However, body weight increased significantly on days 14, 21, and 28 after initiating the RRL treatment (*p <* 0.05 and *p <* 0.01 *vs.* model group). These results suggest that RRL increased the body weight of rats with PF.

### 2.4. RRL Attenuates HYP Expression and Increases GSH and T-SOD Activities in BLM-Treated Rats

**Figure 4:**
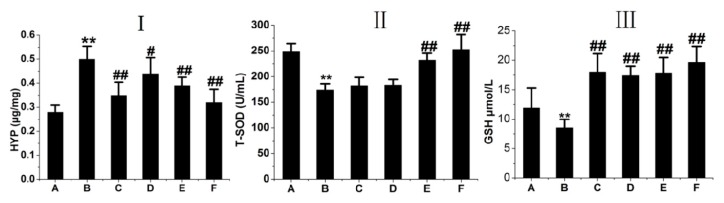
(**I**) lung tissue HYP (hydroxyproline) level in rats; (**II**) serum T-SOD (Total superoxide dismutase) level in rats; (**III**) serum GSH (glutathione hormone) level in rats. (A) normal group; (B) model group; (C) PAG group; (D–F) are RRL groups treated with 125, 250 and 500 mg/kg, respectively. Compared with the Model group. ** *p <* 0.01. Compared with control group, ^#^ *p <* 0.05; ^##^ *p <* 0.01.

HYP is the main component in extracellular collagen and a hallmark of PF [19]. HYP content in the lungs of rats was determined to determine the effect of RRL on lung collagen content. HYP content increased significantly in BLM-treated rats compared to that in the normal group of rats after 28 days, whereas HYP content in the RRL group was significantly lower than that in the model group (Figure 4I). These results indicate that RRL lowered lung collagen content.

Oxidative stress contributes to various pathological conditions and diseases, and BLM promotes oxidative stress damage. BLM-induced oxidative injury was determined by measuring antioxidants, such as T-SOD and GSH, in rat serum. As a result, T-SOD and GSH decreased in serum and BLM-treated lung tissues, whereas they increased after RRL administration.

As shown in Figure 4II,III, serum GSH and T-SOD levels decreased significantly in the model group (T-SOD: 240.24 ± 24.25 U/mL protein in the normal group *vs.* 171.84 ± 25.73 U/mL protein in the model group; GSH: 11.92 ± 3.39 *vs.* 8.53 ± 1.44 μmol/L protein). GSH and T-SOD activities in the RRL group were significantly higher than those in the model group. GSH concentration increased after the RRL (125, 250, and 500 mg/kg) treatments. Oral administration of 250 or 500 mg/kg RRL significantly enhanced T-SOD levels compared with those in the model group (*p <* 0.01, *p <* 0.05). These results show that RRL has antioxidative properties in BLM-induced lung lesions.

### 2.5. RRL Attenuates BLM-Induced *α*-SMA, MMP-9, and TIMP-1 Expression in Rats

α-SMA, MMP-9, and TIMP-1 were immunolocalized as brown DAB precipitates in cells from lung fibrotic regions after 28 days. We observed significant inhibition of α-SMA, MMP-9, and TIMP-1 expression in the RRL-treated rats compared to that in BLM-treated animals after 28 days. MMP-9, TIMP-1, and α-SMA staining density was significantly lower in the RRL-treated rats than that in the model group.

**Figure 5:**
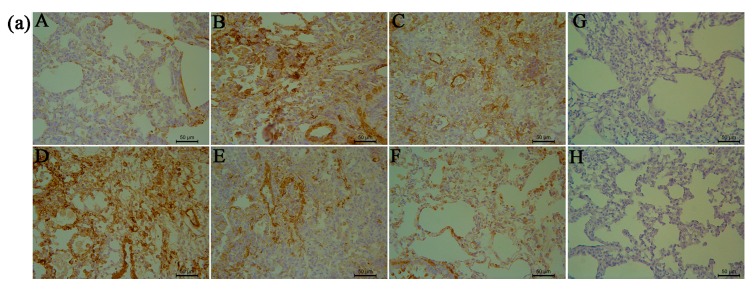

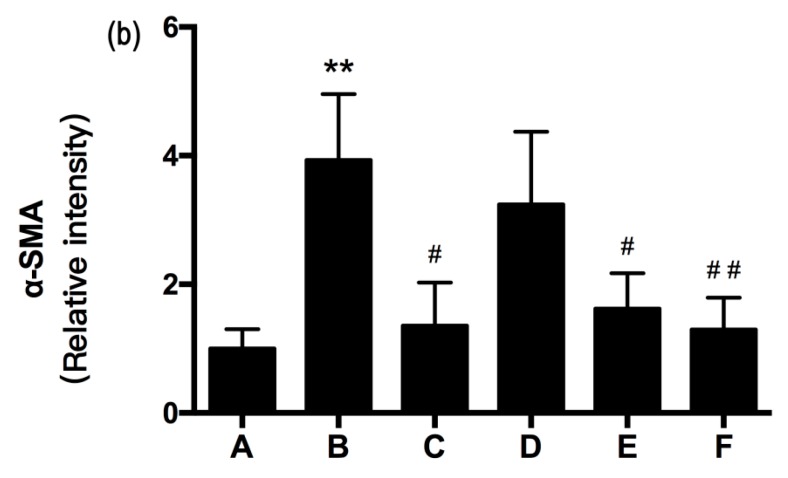
Effects of RRL on the levels of α-smooth muscle actin (α-SMA) in the lung tissues after bleomycin (BLM)-induced in rats. (**a**) representative immunohistochemistry image (Bar = 50 μm). (**A**) normal group; (**B**) model group; (**C**) PAG group; (**D**–**F**) are RRL groups treated with 125, 250 and 500 mg/kg, respectively; (**G**) negative control of omitted first antibody; (**H**) negative control of omitted second antibody; (**b**) the quantitative analysis of α-SMA protein in lung tissues. Data represent the mean ± standard deviation (SD) (*n* = 3) (** *p <* 0.01 *vs.* normal group, ^#^ *p <* 0.05, ^##^ *p <* 0.01, *vs.* model group).

**Figure 6:**
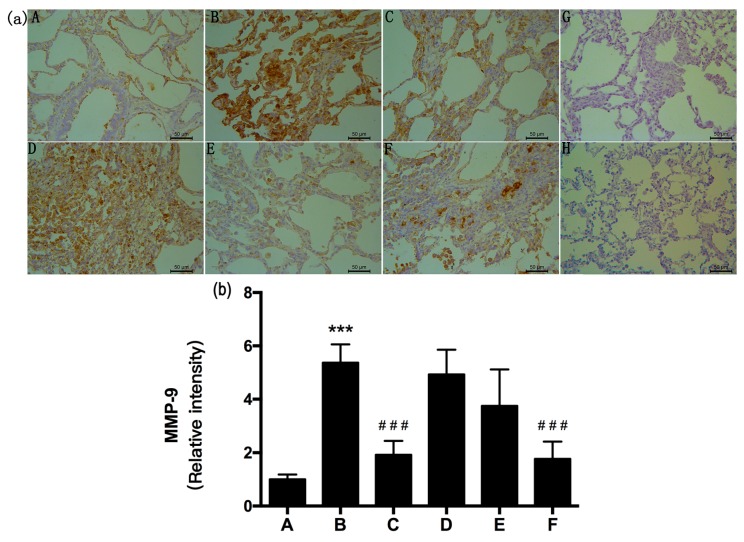
Effects of RRL on the levels of matrix metalloproteinase-9 (MMP-9) in the lung tissues after bleomycin (BLM)-induced in rats. (**a**) representative immunohistochemistry image (Bar = 50 μm). (**A**) normal group; (**B**) model group; (**C**) PAG group; (**D**–**F**) are RRL groups treated with 125, 250 and 500 mg/kg, respectively; (**G**) negative control of omitted first antibody; (**H**) negative control of omitted second antibody; (**b**) the quantitative analysis of MMP-9 protein in lung tissues. Data represent the mean ± standard deviation (SD) (*n* = 3) (*** *p <* 0.001 *vs.* normal group, ^###^ *p <* 0.001 *vs.* model group).

**Figure 7:**
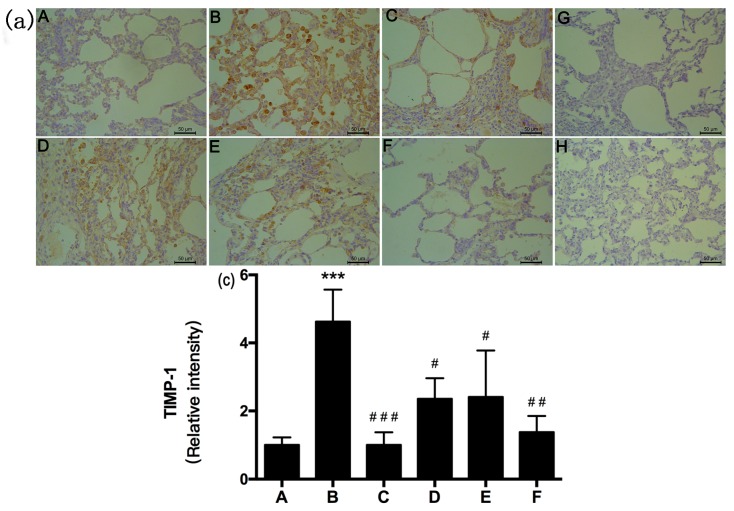
Effects of RRL on the levels of tissue inhibitors of metalloproteinase-1 (TIMP-1) in the lung tissues after bleomycin (BLM)-induced in rats. (**a**) representative immunohistochemistry image (Bar = 50 μm). (**A**) normal group; (**B**) model group; (**C**) PAG group; (**D**–**F**) are RRL groups treated with 125, 250 and 500 mg/kg, respectively; (**G**) negative control of omitted first antibody; (**H**) negative control of omitted second antibody; (**b**) the quantitative analysis of TIMP-1 protein in lung tissues. Data represent the mean ± standard deviation (SD) (*n* = 3) (*** *p <* 0.001 *vs.* normal group, ^#^ *p <* 0.05, ^##^ *p <* 0.01, ^###^ *p <* 0.001 *vs.* model group).

**Figure 8:**
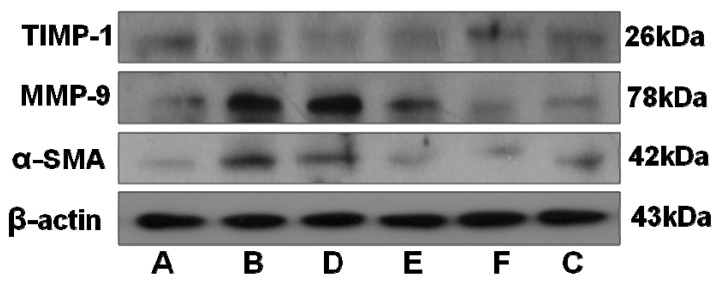
Expression level of TIMP-1, MMP-9 and α-SMA of the lung tissues in rats. (**A**) normal group; (**B**) model group; (**C**) PAG group; (**D**–**F**) are RRL groups treated with 125, 250 and 500 mg/kg, respectively.

As shown in Figure 5, Figure 6 and Figure 7, BLM induced marked increases in α-SMA, MMP-9, and TIMP-1 expression. In contrast, RRL significantly repressed over-expression of α-SMA, MMP-9, and TIMP-1. Consistent with the immunohistochemical staining results, Western blot also revealed that α-SMA and MMP-9 expression in the lungs of rats treated with RRL decreased significantly in a dose-dependent manner. Immunohistochemical staining of lung sections showed that RRL significantly decreased secretion of TIMP-1 in rats with BLM-induced PF (*p <* 0.05, *p <* 0.01, *p <* 0.001 *vs.* model group; Figure 7a,b and Figure 8), but no significant differences were detected by Western blot. Taken together, these results clearly show that RRL reduced α-SMA and MMP-9 expression in the lungs of rats. Therefore, we conclude that the RRL extract probably mitigated α-SMA and MMP-9 expression in rats with BLM-induced PF.

We carried out immunohistochemical staining for α-SMA to investigate proliferation and migration of smooth muscle cells, which contribute to PF pathogenesis. The negative-control (no primary antibody) histological sections did not show any brown staining (Figure 5G). α-SMA is a marker of myofibroblast activation from quiescent lung interstitial fibroblasts. As shown in Figure 5b, BLM induced a marked increase in α-SMA expression (*p <* 0.01), whereas α-SMA expression in rats treated with RRL was significantly lower than that in the model group and decreased dose-dependently after RRL administration.

PF is a cryptic, chronic disease, resulting in progressive and severe pulmonary insufficiency, but the pathogenesis remains poorly understood. The main features of PF include fibroblast proliferation and accumulation of ECM components. The molecular mechanisms responsible for the aberrant tissue remodeling likely involve MMPs and TIMPs, which are the main mediators of ECM turnover.

The negative control (no primary antibody) histological sections for MMP-9 and TIMP-1 immunohistochemistry showed no brown staining (Figure 6G and Figure 7G). Weak MMP-9 and TIMP-1 expression was observed in the organs surrounding the lungs in rats in the normal group. The model group showed significantly increased expression of MMP-9 and TIMP-1 (*p <* 0.001 *vs.* normal group), compared to that in the normal group. Rats in the model group had a broad distribution of MMP-9 and TIMP-1 in bronchial subepithelial tissue and pulmonary interstitial and perivascular spaces. MMP-9 and TIMP-1 expression levels in the RRL group were lower than those in the model group and decreased dose-dependently after RRL administration (Figure 6 and Figure 7). Consistent with the immunohistochemical staining results, Western blot also revealed a suppressive effect of RRL on MMP-9 expression (Figure 8). Taken together, we inferred that RRL reduced α-SMA and MMP-9 expression levels, which slightly ameliorated BLM-induced PF.

### 2.6. RRL Attenuated BLM-Induced TGF-β1 Expression in Rats

**Figure 9:**
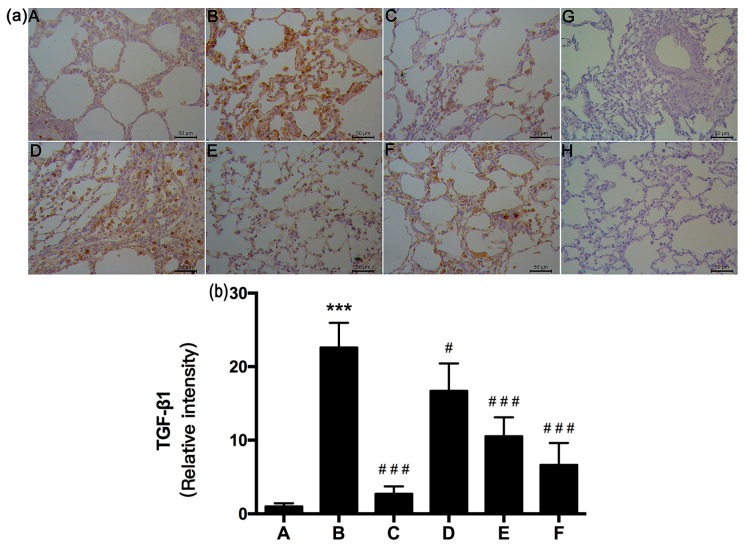
Effects of RRL on the levels of TGF-β1 in the lung tissues after bleomycin (BLM)-induced in rats. (**a**) representative immunohistochemistry image (Bar = 50 μm). (**A**) normal group; (**B**) model group; (**C**) PAG group; (**D**–**F**) are RRL groups treated with 125, 250 and 500 mg/kg, respectively; (**G**) negative control of omitted first antibody; (**H**) negative control of omitted second antibody. Note the absence of the brown coloration; (**b**) the quantitative analysis of TGF-β1 protein in lung tissues. Data represent the mean ± standard deviation (SD) (*n* = 3) (*** *p <* 0.001 *vs.* normal group, ^#^ *p <* 0.05, ^###^ *p <* 0.001 *vs.* model group).

TGF-β1 is the major pro-fibrotic growth factor in lung fibrosis and one of the most widely studied pro-fibrotic cytokines [20,21,22]. TGF-β1 is overexpressed in the lungs of mice during the development of severe interstitial and pleural fibrosis, consisting of excess collagen deposition and the presence of myofibroblasts. Our results show that TGF-β1 was immunolocalized as a brown DAB precipitate in cells from lung fibrotic regions after 28 days. The negative control (no primary antibody) had no brown staining (Figure 9G). TGF-β1 expression increased significantly in the model group (*p <* 0.001; Figure 9a,b), whereas it decreased significantly in RRL-treated rats compared to that in BLM-treated animals after 28 days. TGF-β1 staining intensity in RRL-treated rats was significantly lower than that observed in the model group (*p <* 0.05; *p <* 0.001). These results show that RRL blocked BLM-induced TGF-β1 signaling transduction in lung tissues.

### 2.7. Small Molecule Compounds

We identified the structures of nine small molecule compounds in RRL, and nine components were identified in the RRL by NMR, UV, and IR (Table 2).

HMBC, heteronuclear multiple-bond correlation; HSQC, heteronuclear singular quantum correlation; NOESY, nuclear overhauser effect spectroscopy; m.p., melting point; UV, ultraviolet spectrum; CD, circular dichroism spectrum; HR-ESI-TOF-MS, high-resolusion electrospray-ionization time-of-flight mass spectrometry; TLC, thin-layer chromatography.

### 2.8. Characterization of the RRL Constitutents by UPLC-TOF-MS

UPLC-TOF-MS was employed to analyze the RRL crude extracts. Nine components were identified by NMR, UV, and IR by comparing the exact masses and formulae from their MS data by UPLC-MS. Among them, seven compounds showed satisfactory binding with FAPα, based on the bioinformatics analysis results described above, including 3,4,5-trihydroxybenzoic acid (2*S*-*cis*)-3,4-dihydro-5,7-dihydroxy-2-(3,4,5-trihydroxyphenyl)-2*H*-benzo[*b*]pyran-3-yl ester (**1**), herbacetin-3-*O*-β-d-glucopyranoside-7-*O*-α-l-rhamnoside (**2**), kaempferol-7-*O*-α-l-rhamnoside (**3**), Rhodiocyanoside A (**4**), Rosarin (**5**), Rosavin (**6**), and Rhodionin (**7**) (Table 3).

### 2.9. Molecular Docking of FAP-α

**Figure 10:**
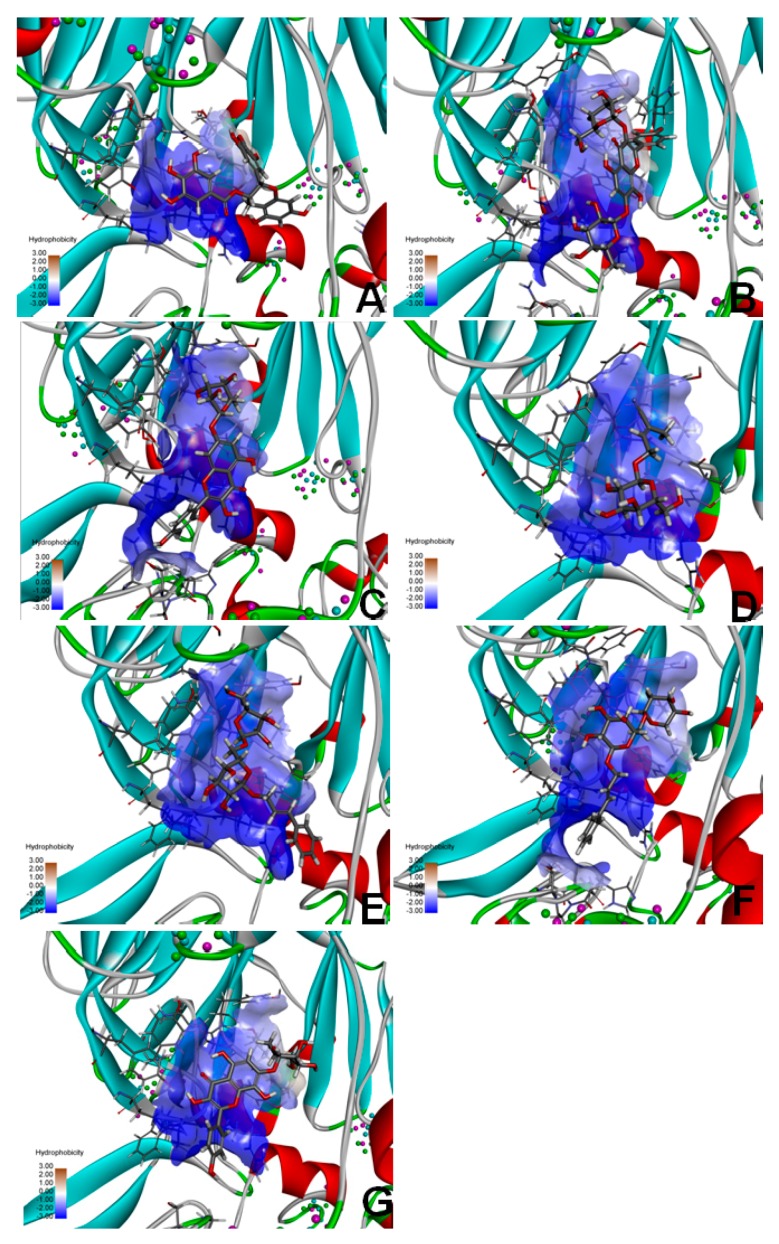
The docking results of the seven compounds interacting with the target protein fibroblast activation protein-α (FAP-α). (**A**) 3,4,5-trihydroxybenzoic acid (2*S*-*cis*)-3,4-dihydro-5,7-dihydroxy -2-(3,4,5-trihydroxyphenyl)-2*H*-benzo[*b*]pyran-3-yl ester; (**B**) Herbacetin-3-*O*-β-d-glucopyranoside-7-*O*-α-l-rhamnoside; (**C**), Kaempferol-7-*O*-α-l-rhamnoside; (**D**) Rhodiocyanoside A; (**E**) Rosarin; (**F**) Rosavin; (**G**) Rhodionin.

To identify the targets related to the preventive effects of RRL on BLM-induced PF, the seven compounds from RRL were selected for molecular docking targeting FAP-α to determine their effect on PF in rats. The molecular docking results were ranked by a score indicating the degree of interaction with FAP-α, hence indicating their effectiveness for treating PF (Figure 10). The list is as follows: 3,4,5-trihydroxybenzoic acid (2*S*-*cis*)-3,4-dihydro-5,7-dihydroxy-2-(3,4,5-trihydroxyphenyl)-2*H*-benzo[*b*]pyran-3-yl ester (Figure 10A); Herbacetin-3-*O*-β-d-glucopyranoside-7-*O*-α-l-rhamnoside (Figure 10B); Kaempferol-7-*O*-α-l-rhamnoside (Figure 10C); Rhodiocyanoside A (Figure 10D); Rosarin (Figure 10E); Rosavin (Figure 10F); and Rhodionin (Figure 10G).

## 3. Discussion

The process of PF is complex, and the specific mechanism remains unclear. In this study, a rat model of PF was established by intratracheal injection of BLM [23]. BLM forms BLM-Fe^2+^ complexes, which provide electrons to oxygen molecules to form peroxides and hydroxyl radicals, leading to free radicals in deoxyribose of DNA. A large number of free radicals in lung tissue causes damage by lipid peroxidation and inflammation during early PF, which further promotes proliferation of fibroblasts and alveolar-interstitial fibrosis [24]. The results of the present study show a significant increase in lung HYP content but decreased GSH content, T-SOD activity, and inflammatory factors in BAL, such as TNF-α, TGF-β1, and IL-6. In addition, the alveoli of the rats treated with BLM were structurally disordered, with obvious alveolitis, numerous alveolar septal fibroblasts, and ECM deposits typical of PF. The immunohistochemical results showed that α-SMA, MMP-9, and TGF-β1 expression increased in fibrotic lung tissue. These results suggest that the BLM-induced rat PF model was successfully established.

An important pathological feature of PF is excessive deposition of ECM, and HYP is a characteristic component of collagen. Therefore, we considered HYP as a PF indicator [25]. Our results show that HYP content in the lungs of BLM-treated rats was significantly higher than that in the normal group, whereas lung HYP content in the RRL-treated group was significantly lower than that in the BLM group, suggesting that the protective effects of RRL on BLM-induced PF were highly associated with decreased lung HYP content. We showed that RRL has powerful antioxidant capacity. Serum GSH and T-SOD activities increased significantly in the RRL-treated groups compared with those in the BLM group (*p <* 0.01), indicating clearance of reactive oxygens species, and enhanced antioxidant capacity, which reduced peroxidation of oxygen free radicals and damage to cells and tissues.

TGF-β1 is a key fibrogenic cytokine that binds to receptors on fibroblasts and activates the TGF-β/Smads signaling pathway. Tanjore *et al.* demonstrated that fibroblasts differentiate into an ECM-producing myofibroblast phenotype when tissues become fibrotic, which further exacerbates tissue stiffness and injury [26,27]. However, the metabolic disorder in the ECM during PF may cause excessive deposition of ECM, subsequently leading to PF. MMPs and TIMPs normally maintain dynamic equilibrium in the ECM. MMP-9 is involved in degrading and reconstructing the ECM, and enhanced MMP and decreased TIMP activities undermine homeostasis of the ECM. TGF-β1 regulates MMP-9 and TIMP-1 expression and the metabolic imbalance in the ECM, which reduces ECM deposition in the stroma, to mitigate and contain the development of PF. Moreover, TGF-β1 enhances phosphorylation of Smad2 and Smad3 in cells, leading to the TGF-β-dependent EMT and subsequent development and progress of fibrosis-related lung diseases [28,29]. We speculate based on previous studies, and our results, that RRL would inhibit phosphorylation of Smad3 caused by the decrease in TGF-β1. However, the actual effect of RRL on p-Smad3 expression requires further study, which may more clearly elucidate the underlying mechanisms of the RRL-induced protective effect on PF.

In this study, the pathological observations in lung tissue showed that rats treated with the high dose of RRL had less lung inflammation and fibrosis than those in BLM-treated rats. We also determined that RRL significantly reduced α-SMA and MMP-9 expression in the BLM-induced PF rat model. Consistently, this study showed that expression of MMP-9 increased significantly while TGF-β1 was inhibited in the lungs of rats. These results strongly suggest that RRL attenuated BLM-induced fibrotic lung injury in rats.

## 4. Materials and Methods

### 4.1. Reagents

The roots and rhizomes of *Rhodiola rosea* L. (RRL) were collected in Tacheng Xinjiang of China in October 2007 and identified by Yong Tan (Shihezi University, China). A voucher specimen (No. 20071003001) was deposited in the School of Pharmacy, Shihezi University. Bleomycin (BLM) and prednisone acetate were purchased from Nippon Kayaku (Tokyo, Japan) and Tianjin Lisheng Pharmaceutical Co., Ltd. (Tianjin, China), respectively. Hydroxyproline (HYP; Nanjing Jiancheng Biological Engineering Research Institute, Nanjing, China), glutathione (GSH; Nanjing Jiancheng Biological Engineering Research Institute), and total superoxide dismutase (T-SOD; Nanjing Jiancheng Bioengineering Institute) were purchased commercially. Enzyme-linked immunosorbent assay (ELISA) kits for rat TNF-α (Shanghai Xitang, Shanghai, China), IL-6 (Shanghai Xitang), and TGF-β1 (Shanghai Xitang) were used according to the manufacturer’s instructions. Mouse anti-α-SMA monoclonal antibody (Boster Biological Technology, Pleasanton, CA, USA), rabbit anti-MMP-9 polyclonal antibody, rabbit anti-TGF-β1 polyclonal antibody (Boster Biological Technology) and horseradish peroxidase (HRP)-labeled goat anti-rabbit IgG antibody (Boster Biological Technology) were purchased and used as received. RIPA (Radio Immunoprecipitation Assay) lysis buffer (Solarbio Biological Technology, Beijing, China), BCA (Bicinchoninic acid) Protein Assay Kit (Solarbio Biological Technology), HRP-conjugated secondary antibodies (Boster Biological Technology), and an ECL (Enhanced Chemiluminescent) detection kit (Beyotime, Haimen, China) were also obtained.

### 4.2. Preparation of RRL Extract

The air-dried roots and rhizomes of RRL were refluxed three times with 95% ethanol (1:10) (2 h × 3). The combined 95% ethanol extracts were concentrated in a vacuum at 45 °C to generate a crude extract.

### 4.3. Ultra High Pressure Liquid Chromatography Time-of-Flight Mass Spectrometry (UPLC-TOF-MS) Analysis

The analytical procedure was performed using a system consisting of a Waters Acquity UPLC (Ultra Performance Liquid Chromatography) and Waters LCT Premier XE time-of-flight mass spectrometer (Waters Inc., Milford, MA, USA). Chromatography was performed using an ACQUITY UPLC^®^ BEH C18 column (2.1 × 50 mm, 1.7 μm), and column temperature was maintained at 30 °C. The mobile phase (flow rate, 0.2 mL/min) consisted of solvent A (1% formic acid) and solvent B (50:50 menthol: acetonitrile with 1% formic acid), using the following gradient elution profile: 0–7 min, 9%–20% A; 7–18 min, 20%–22% A; 18–25 min, 22%–50% A; and 25–27 min; and 50%–95% B. The mass spectrometer was optimized in ±V mode, source temperature was set to 100 °C with cone gas flow of 30 L/h, desolvation gas temperature was 300 °C, desolvation gas flow was 650 L/h, capillary voltage was 2200 V, sample cone voltage was 100 V, and extraction cone voltage was 100 V. The data were processed using Masslynx 4.1 software (Waters Inc.).

### 4.4. Animals

Male Sprague–Dawley (SD) rats (weight, 200 ± 20 g) were obtained from the Animal Center of Urumqi (Certificate No. SCXK [xin] 2011-0003, Urumqi, China) and maintained on a 12-h light/dark cycle at a controlled temperature with free access to food and tap water. Animal welfare and the experimental procedures were in accordance with the Ethical Regulations on the Care and Use of Laboratory Animals of Shihezi University, and all animal experiments were performed with the approval and under the guidelines of the Animal Experimental Ethics Committee of the First Affiliated Hospital of medical college, Shihezi University (A2012-016, 30 May 2012).

### 4.5. BLM-Induced PF Rat Model

Forty-eight male SD rats were divided randomly into the following six groups: normal control, model (BLM group), prednisone acetate control (PAG group, 3.34 mg/kg), and three groups received oral RRL (125, 250, and 500 mg/kg). BLM was used to induce PF. Briefly, the rats were anesthetized with an injection of 10% chloride hydrate (3.5 mL/kg) and administered a single intratracheal instillation of saline containing 5 mg/kg BLM [30]. The rats were rotated immediately to ensure a thorough drug distribution in the lungs. Rats in the control group underwent the same surgical procedure but were given an equal volume of saline instead of BLM (5 mg/kg). Thereafter, the rats in the control and BLM groups were administered saline (10 mL/kg body weight) intragastrically once daily. RRL (10 mL/kg body weight) was dissolved in saline and administered once by oral gavage. The positive control group was treated with the same volume of PAG in saline. The body weights of the rats were monitored weekly, and all rats were killed after 28 days. Blood samples were collected from the abdominal artery, centrifuged at 4000 rpm for 10 min at 4 °C to obtain the serum, and stored at −80 °C before assay. Then, bronchoalveolar lavage fluid (BALF) was collected by intratracheal instillation and drainage of 5 mL phosphate buffered saline (PBS) three times. Half of the left lung tissue was removed and fixed in 4% paraformaldehyde, and the other half was frozen to prepare the homogenate.

### 4.6. Lung Collagen Content

Lung collagen content was evaluated using an HYP kit according to the manufacturer’s protocol. Briefly, 50 mg of frozen lung tissue sample was hydrolyzed in 1 mL lysis buffer at 95 °C for 20 min, and absorbance was measured at 550 nm. Each sample was run in triplicate. Collagen content in pulmonary tissues was expressed as micrograms of hydroxyproline per gram wet lung weight (µg/g) [31,32].

### 4.7. Antioxidant Enzyme Activities

T-SOD and GSH activities were determined using kits according to the manufacturer’s instructions. T-SOD data are expressed as U/mL. The GSH reaction was measured at 405 nm, and enzyme activity is presented as μmol/L.

### 4.8. BALF Biochemical Analysis

The BALF was centrifuged at 1500 rpm for 10 min at 4 °C, and the supernatant was stored immediately at −80 °C. BALF levels of TNF-α, TGF-β1, and IL-6 were determined using commercially available ELISA kits according to the manufacturer´s instructions. The optical density value was determined at 450 nm using an ELISA reader and calculated at the linear portion of the curve. The data are presented as picograms per milliliter (pg/mL) BALF.

### 4.9. Histopathological Examination

Lung specimens were fixed in 10% formalin and embedded in paraffin. The paraffin-embedded specimens were sectioned into 5-μm thick slices, deparaffinized in xylene for 15 min, hydrated in a graded alcohol series, and rinsed three times with 1% PBS. The slices were stained with hematoxylin and eosin (H & E) and Masson’s trichrome for microscopic observations according to the manufacturer’s standard protocols. Masson’s trichrome stain was used to show collagen deposition, as collagen fibers stain blue. Nuclei stained dark red/purple, and the cytoplasm stained red/pink. The sections were examined under a Nikon 80i plus confocal laser-scanning microscope (Nikon, Tokyo, Japan) at ×100 magnification.

### 4.10. Immunohistochemical Staining

MMP-9, TIMP-1, TGF-β1, and α-SMA expression levels in the lung were detected by immunohistochemistry. The sections (5-μm) were dewaxed, rehydrated, washed in PBS, and endogenous peroxidase was inactivated with 3% H_2_O_2_ for 15 min at room temperature. After washing three times with PBS, the sections were blocked with normal goat serum for 30 min at 37 °C and incubated with primary antibodies against MMP-9 (1:200), TIMP-1 (1:100), TGF-β1 (1:100), and α-SMA (1:200) overnight at 4 °C. After washing the slides three times with PBS, the sections were incubated with secondary peroxidase-conjugated affinity pure goat anti-rabbit IgG antibody (H+L) (1:50; ZSGB-BIO, Beijing, China) for 20 min at 37 °C. The sections were washed with PBS and incubated for 15 min with the streptavidin-biotin-peroxidase complex. After washing with PBS, diaminobenzidine (DAB) was added as a visualizing agent. The nuclei were counterstained with hematoxylin. A negative control was created by omitting the primary or secondary antibody. The sections were examined under a Nikon 80i plus confocal laser-scanning microscope. Each sample was tested in triplicate. The number of positively stained cells in each tissue section was calculated from a 50 μm magnified field under a light microscope. For immunohistochemical analyses of MMP-9, TIMP-1, TGF-β1, and α-SMA, staining density was determined using Image Pro plus 6.0 software (Media Cybernetics, Silver Spring, MD, USA) in one field of each section with a prominent DAB reaction.

### 4.11. Western Blotting

Tissues were collected from each treatment and suspended in RIPA lysis buffer. The lysed tissue was centrifuged at 12,000× *g* for 10 min, the supernatant was collected, and protein content was determined using a BCA Protein Assay Kit. The proteins were separated by 12% sodium dodecyl sulfate–polyacrylamide gel electrophoresis and transferred to a PVDF (polyvinylidene fluoride) membrane (Millipore Corp., Billerica, MA, USA). The membranes were blocked with 5% nonfat milk in TBST (Tris-Buffered Saline and Tween 20) and the target proteins were incubated overnight with primary antibodies against α-SMA (1:500) and MMP-9 (1:500). After four 10-min washes with TBST, the membranes were incubated with HRP-conjugated secondary antibodies (1:1000) for 2 h at room temperature. The blots were washed four times in TBST buffer, subjected to an ECL detection kit, and exposed to photographic film. β-Actin served as the internal standard for protein loading and transfer.

### 4.12. Isolation and Identification of the Main Components in Rhodiola rosea L.

We isolated 10 compounds and identified their structures by applying silica gel (200–300 mesh; Qingdao Marine Chemical Group Co., Qingdao, China), Sephadex LH-20 (General Electric, Fairfield, CA, USA), ODS (30–50 μm; YMC Co., Ltd., Tokyo, Japan), and UPLC-TOF-MS. In brief, the dried RRL roots and rhizomes (2.0 kg) were refluxed three times with EtOH:H_2_O (95:5, *v*/*v*, 20 L). The ethanol extract was harvested by vacuum concentration (340 g). The extract was suspended in water (2.0 L) and extracted stepwise with petroleum ether (PE), CHCl_3_, EtOAc, and *n*-BuOH (3 × 2.0 L) to obtain PE-soluble (8.0 g), CHCl_3_-soluble (16 g), EtOAc-soluble (56 g), and *n*-BuOH-soluble fractions (204 g). The EtOAc-soluble fraction (50 g) was subjected to silica gel column chromatography (CC) and eluted with CHCl_3_ containing an increasing volume of MeOH to afford 15 fractions (A–O: 100:0–0:100). Fraction E (CHCl_3_–MeOH, 100:4.5) (1.2 g) was applied to silica gel CC, eluted with CHCl_3_:MeOH (4:1), and isolated with absolute MeOH by Sephadex LH-20 CC to produce compound **3** (45 mg). Fraction F-1 (CHCl_3_:MeOH, 100:6) (2.8 g) was applied to Sephadex LH-20 and eluted with MeOH:H_2_O (50:50) to produce compound **7** (42 mg). Fraction F-2 (CHCl_3_:MeOH, 100:6) (1.8 g) was applied to silica gel CC and eluted with CHCl_3_:MeOH (100:8) to produce compound **1** (52 mg).

An aliquot of the *n*-BuOH-soluble fraction (180 g) was subjected to silica gel CC using a CHCl_3_:MeOH gradient to afford 16 fractions (A–P: 100:0–0:100). Fraction B (CHCl_3_:MeOH, 100:1) (1.8 g) was separated via HPLC, using MeOH:H_2_O (35:65, *v*/*v*) as the mobile phase to produce compound **4** (300 mg). Fraction F (CHCl_3_:MeOH, 100:6) (3.2 g) was separated via HPLC with MeOH:H_2_O (23:77, *v*/*v*) as the mobile phase to produce compound **5** (100 mg). Fraction G-1 (CHCl_3_:MeOH, 100:8) (2 g) was separated via HPLC with MeOH:H_2_O (32:68, *v*/*v*) as the mobile phase to produce compound **6** (480 mg). Fraction G-1 (CHCl_3_:MeOH, 100:8) (3.3 g) was separated via HPLC with MeOH:H_2_O (35:65, *v*/*v*) as the mobile phase to produce compound **2** (82 mg). The structures of compounds **1**–**7** were identified by comparing their spectroscopic data with those reported in the literature [33,34,35,36,37]. The relevant chromatograms and mass spectral data of the active compounds are as follows.

Compound **1**: colorless needle (MeOH), m.p. 192.6–193.6 °C ultraviolet (UV) (MeOH) λ_max_ (log ε) nm: 210 (4.93), 276 (4.10). CD (MeOH, *c* = 0.00552 g/100 mL) λ_max_ (θ) nm (deg·cm^2^·dmol^−1^): 214 (−788), 242 (−18), 278 (−210). Infrared (IR) (KBr) ν (cm^−1^): 3172, 2875, 1699, 1619, 1532, 1457, 1314, 1227, 1133, 1021, 823. ESI-TOF-MS: 459.0931 [M + H]^+^ (Calcd. for C_22_H_19_O_11_ 459.0927, 0.9 ppm), 917.1791 [2M + H]^+^ (Calcd. for C_44_H_37_O_22_ 917.1776, 1.6 ppm), 1375.2662 [3M + H]^+^ (Calcd. for C_66_H_55_O_33_ 1375.2626, 2.6 ppm); ^1^H-nucelar magnetic resonance (NMR) (CD_3_OD, 600 MHz) δ: 4.96 (br. s. H-2), 5.36 (br. s. H-3), 2.66 (d, *J* = 16.8 Hz, H-4) and 2.93 (dd, *J* = 16.8, 4.8 Hz, H-4), 5.83 (d, *J* = 2.4 Hz, H-5), 6.82 (2H, s, H-2′, 6′), 8.07 (br.s, OH), 8.74 (3H, br.s, OH), 9.07 (2H, br.s, OH), 9.20 (2H, br.s, OH), 9.31 (2H, br.s, OH); ^13^C-NMR (CD_3_OD, 150 MHz) δ: 76.4 (C-2), 70.0 (C-3), 25.7 (C-4), 156.5 (C-5, 9), 94.3 (C-6), 155.6 (C-7), 95.5 (C-8), 97.3 (C-10), 128.6 (C-1′), 105.4 (C-2′, 6′), 145.4 (C-3′, 5′), 132.3 (C-4′), 165.2 (C-1′′), 119.2 (C-2′′), 108.6 (C-3′′, 7′′), 145.6 (C-4′′, 6′′), 138.5 (C-5′′).

Compound **2**: yellow powder (MeOH), HR-ESI-TOF-MS: 609.1471 [M − H]^−^ (Calcd. for C_27_H_29_O_16_ 609.1456, 2.5 ppm); ^1^H-NMR (300 MHz, DMSO-*d*_6_) δ: 12.03 (1H, s, 5-OH), 10.22 (1H, s, 4′-OH), 8.85 (1H, s, 8-OH), 8.14 (2H, d, *J* = 8.7 Hz, H-2′, 6′), 6.90 (2H, d, *J* = 8.7 Hz, H-3′, 5′), 6.61 (1H, s, H-6), 5.46 (1H, d, *J* = 6.6 Hz, glc-1), 5.50 (1H, br.s, rha-1), 1.11 (3H, d, *J* = 6.0 Hz, rha-CH_3_), 4.70~5.50 (4H, br.s, glc-2, 3, 4, 6-OH), 3.18~4.52 (6H, m, glc-2, 3, 4, 5, 6), δ 4.70~5.50 (3H, br.s, rha-2, 3, 4-OH), 3.18~3.96 (4H, m, rha-2, 3, 4, 5); ^13^C-NMR (75 MHz, DMSO-*d*_6_) δ: 157.1 (C-2), 133.3 (C-3), 177.5 (C-4), 152.2 (C-5), 99.4 (C-6), 150.8 (C-7), 144.9 (C-8), 127.2 (C-9), 105.6 (C-10), 121.1 (C-1′), 131.2 (C-2′, 6′), 160.2 (C-4′), 115.1 (C-3′,5′), 101.5 (glc-1), 74.3 (glc-2), 76.5 (glc-3), 70.8 (glc-4), 77.6 (glc-5), 61.0 (glc-6), 98.9 (rha-1), 70.6 (rha-2), 70.0 (rha-3), 71.8 (rha-4), 70.0 (rha-5), 18.0 (rha-6).

Compound **3**: yellow powder (MeOH), m.p. 231.3–233.8 °C, HR-ESI-TOF-MS: 433.1137 [M + H]^+^ (Calcd. for C_21_H_21_O_10_ 433.1135 0.5 ppm); ^1^H-NMR (600 MHz, CD_3_OD) δ: 8.10 (2H, d, *J* = 8.4 Hz, H-2′, 6′), 7.33 (2H, d, *J* = 8.4 Hz, H-3′, 5′), 6.74 (1H, d, *J* = 2.4 Hz, H-8), 6.42 (1H, d, *J* = 1.8 Hz, H-6), 5.56 (1H, br.s, *J* = 2.4 Hz, rha-1), 3.84 (1H, dd, *J* = 3.6, 3.0 Hz, rha-2), 4.02 (1H, m, rha-3), 3.48 (1H, t, *J* = 9.6, 9.0 Hz, rha-4), 3.61 (1H, m, rha-5), 1.26 (3H, d, *J* = 6.0 Hz, rha-6); ^13^C-NMR (150 MHz, CD_3_OD) δ: 148.7 (C-2), 137.5 (C-3), 177.5 (C-4), 163.3 (C-5), 99.8 (C-6), 162.3 (C-7), 95.3 (C-8), 157.7 (C-9), 106.2 (C-10), 122.1 (C-1′), 130.8 (C-2′, 6′), 160.8 (C-4′), 116.4 (C-3′, 5′), 99.9 (rha-1), 71.7 (rha-2), 73.6 (rha-3), 71.2 (rha-4), 72.1 (rha-5), 18.1 (rha-6).

Compound **4**: colorless glassy substance (MeOH), HR-ESI-TOF-MS: 282.0951 [M + Na]^+^ (Calcd. for C_11_H_17_NO_6_Na 282.0954, −1.1 ppm); ^1^H-NMR (600 MHz, DMSO-*d*_6_) δ: 6.50 (1H, td, *J* = 1.8, 6.6, 7.2 Hz, H-3), 4.42 (1H, dd, *J* = 1.8, 6.6 Hz, H-4), 4.42 (1H, dd, *J* = 6.6, 7.2 Hz, H-4), 1.95 (3H, s, 2-CH_3_), 4.24 (1H, d, *J* = 7.8 Hz, glc-1), 4.49~5.07 (4H, br.s, glc-2, 3, 4, 6-OH), 2.94~3.67 (6H, m, glc-2, 3, 4, 5, 6); ^13^C-NMR (150 MHz, DMSO-*d*_6_) δ: 118.1 (C-1), 112.6 (C-2), 145.0 (C-3), 68.4 (C-4), 20.2 (C-5), 103.3 (C-1′), 74.0 (C-2′), 77.6 (C-3′), 70.6 (C-4′), 77.4 (C-5′), 61.6 (C-6′).

Compound **5**: white needles (MeOH), HR-ESI-TOF-MS: 451.1578 [M + Na]^+^ (Calcd. for C_20_H_28_O_10_Na 451.1580, −0.4 ppm); ^1^H-NMR (600 MHz, DMSO-*d*_6_) δ: 7.43 (2H, m, H-2, 6), 7.33 (2H, m, H-3, 5), 7.24 (1H, m, H-4), 6.66 (1H, d, *J* = 15.6 Hz, H-7), 6.35 (1H, dt, *J* = 16.2, 5.4 Hz, H-8), 4.40 (1H, dd, *J* = 13.2, 4.8 Hz, H-9a), 4.20 (1H, dd, *J* = 15.6, 6.0 Hz, H-9b), 4.24 (1H, d, *J* = 7.8 Hz, glc-1′), 3.02~3.95 (6H, m, glc-2′, 3′, 4′, 5′, 6′), 4.52 (1H, d, *J* = 4.8 Hz, Ara-1′′), 3.02~3.91 (5H, m, Ara-2′′, 3′′, 4′′, 5′′); ^13^C-NMR (150 MHz, DMSO-*d*_6_) δ: 136.7 (C-1), 128.8 (C-2, 6), 126.6 (C-3, 5), 126.3 (C-4), 131.8 (C-7), 127.8 (C-8), 68.8 (C-9), 102.1 (C-1′), 73.7 (C-2′), 76.8 (C-3′), 70.6 (C-4′), 75.7 (C-5′), 67.4 (C-6′), 108.8 (C-1′′), 82.4 (C-2′′), 77.5 (C-3′′), 84.0 (C-4′′), 61.6 (C-5′′).

Compound **6**: white needles (MeOH), m.p. 170–172 °C, HR-ESI-TOF-MS: 451.1583 [M + Na]^+^ (Calcd. for C_20_H_28_O_10_Na 451.1580, 0.7 ppm); ^1^H-NMR (600 MHz, DMSO-*d*_6_) δ: 7.46 (2H, m, H-2, 6), 7.34 (2H, m, H-3, 5), 7.24 (1H, m, H-4), 6.68 (1H, d, *J* = 15.6 Hz, H-α), 6.36 (1H, dt, *J* = 16.2, 6.0 Hz, H-β), 4.20~4.23 (2H, dd, H-γ), 4.58 (1H, d, *J* = 15.6 Hz, glc-1′), 3.02–3.95 (6H, m, glc-2′, 3′, 4′, 5′, 6′), 4.52 (1H, d, *J* = 4.8 Hz, Ara-1′′), 3.02~3.95 (5H, m, Ara-2′′, 3′′, 4′′, 5′′); ^13^C-NMR (150 MHz, DMSO-*d*_6_) δ: 136.5 (C-1), 128.6 (C-2, 6), 127.6 (C-3, 5), 126.3 (C-4), 131.5 (C-α), 126.2 (C-β), 68.5 (C-γ), 101.9 (C-1′), 73.4 (C-2′), 76.6 (C-3′), 70.2 (C-4′), 75.7 (C-5′), 64.9 (C-6′), 103.5 (C-1′′), 72.5 (C-2′′), 70.5 (C-3′′), 68.1 (C-4′′), 67.3 (C-5′′).

Compound **7**: yellow powder (MeOH), m.p. 232.8–235.9 °C, HR-ESI-TOF-MS: 449.1085 [M + H]^+^ (Calcd. for C_21_H_21_O_11_ 449.1084, 0.2 ppm); ^1^H-NMR (600 MHz, CD_3_OD) δ: 11.92 (1H, s, 5-OH), 8.12 (2H, d, *J* = 9.0 Hz, H-2′, 6′), 6.92 (2H, d, *J* = 8.4 Hz, H-3′, 5′), 7.05 (1H, s, H-6), 5.56 (1H, d, *J* = 1.2 Hz, rha-1), 7.05 (1H, s, rha-2-OH), 6.74 (2H, d, rha-3-OH), 6.42 (2H, d, rha-4-OH), 3.84 (1H, m, rha-2), 4.02 (1H, m, rha-3), 3.49 (1H, m, rha-4), 3.61 (1H, m, rha-5); ^13^C-NMR (150 MHz, CD_3_OD) δ: 147.5 (C-2), 135.8 (C-3), 176.4 (C-4), 150.1 (C-5), 98.3 (C-6), 151.6 (C-7), 127.1 (C-8), 144.5 (C-9), 104.5 (C-10), 121.8 (C-1′), 129.8 (C-2′, 6′), 159.3 (C-4′), 115.4 (C-3′, 5′), 99.4 (rha-1), 70.0 (rha-2), 69.9 (rha-3), 71.7 (rha-4), 69.9 (rha-5), 17.9 (rha-6).

### 4.13. Molecular Docking

Seven molecular compounds were selected as ligands among the small compounds targeting fibroblast activation protein-α (FAP-α). We downloaded the initial three-dimensional (3D) geometric coordinates of the X-ray crystal structures of the compounds and proteins from the Protein Data Bank [38], and the chemical structures of the candidate compounds were drawn using ChemDraw software ver. 12.0 [39]. The 3D FAP-α protein structure (PDB ID: 1Z68) interacted with the candidate compounds. Molecular docking was performed using the UCSF DOCK6.5 program [40]. The DOCK algorithm addresses rigid body docking by superimposing the ligand onto a negative image of the binding pocket.

### 4.14. Statistical Analysis

All quantitative data are expressed as mean ± standard deviation. Data were analyzed using SPSS 17.0 software (SPSS Inc., Chicago, IL, USA). One-way analysis of variance was used to detect differences among the groups. A *p*-value <0.05 was considered significant.

## 5. Conclusions

In summary, RRL had prophylactic and therapeutic effects on BLM-induced PF in rats, which were related to its anti-inflammatory and anti-oxidative properties. RRL may reduce the expression and activities of MMP-9 and α-SMA. Further study is necessary to elucidate the mechanisms of how RRL reduced MMP-9 expression and activity.

## Figures and Tables

**Table 1:** Changes of body weight after drugs intervention (*n* = 10).

Group	Day 1	Day 7	Day 14	Day 21	Day 28
A	185.3 ± 2.2	209.9 ± 8.5	243.3 ± 13.4	262.5 ± 19.6	294.0 ± 16.6
B	190.7 ± 5.4	190.4 ± 22.2 *	195.0 ± 18.9 **	227.7 ± 21.4 **	236.0 ± 18.5 **
C	186.9 ± 6.5	181.1 ± 15.1	183.8 ± 19.2	255.4 ± 21.9 ^##^	270.3 ± 20.3 ^##^
D	187.3 ± 4.8	192.6 ± 18.0	219.1 ± 9.6 ^##^	253.0 ± 14.3 ^#^	279.3 ± 14.8 ^##^
E	190.9 ± 3.1	196.2 ± 12.0	225.5 ± 12.8 ^##^	273.4 ± 20.8 ^##^	282.0 ± 18.4 ^##^
F	189.6 ± 6.9	197.6 ± 8.9	223.3 ± 8.8 ^##^	272.9 ± 23.7 ^##^	284.6 ± 9.3 ^##^

Value are expressed with mean ± SD (*n* = 10). * *p <* 0.05, ** *p <* 0.01 *vs.* the control group; ^#^ *p <* 0.05, ^##^ *p <* 0.01 *vs.* the model group. A: Normal group; B: Model group; C: PAG group; D–F are RRL group treated with 125, 250 and 500 mg/kg, respectively.

**Table 2:** The structures of compounds in *Rhodiola rosea* L.

No.	Name	Structure	Identification Methods
**1**	3,4,5-trihydroxybenzoic acid (2*S*-*cis*)-3,4-dihydro-5,7-dihydroxy-2-(3,4,5-trihydroxyphenyl)-2*H*-benzo[*b*]pyran-3-yl ester	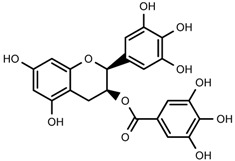	^1^H-NMR, ^13^C-NMR, HMBC, HSQC, CD, IR, UV, HR-ESI-TOF-MS
**2**	herbacetin-3-*O*-β-d-glucopyranoside-7-*O*-α-l-rhamnoside	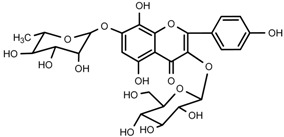	^1^H-NMR, ^13^C-NMR, HMBC, HR-ESI-TOF-MS
**3**	kaempferol-7-*O*-α-l-rhamnoside	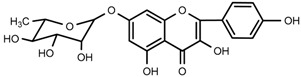	^1^H-NMR, ^13^C-NMR, HR-ESI-TOF-MS, m.p.
**4**	Rhodiocyanoside A	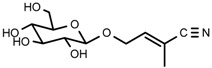	^1^H-NMR, ^13^C-NMR, HR-ESI-TOF-MS
**5**	Rosarin	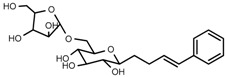	^1^H-NMR, ^13^C-NMR, HR-ESI-TOF-MS
**6**	Rosavin	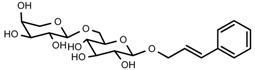	^1^H-NMR, ^13^C-NMR, HR-ESI-TOF-MS
**7**	Rhodionin	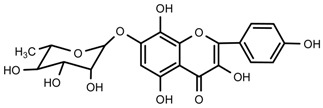	^1^H-NMR, ^13^C-NMR, HR-ESI-TOF-MS, m.p.
**8**	β-sitosterol	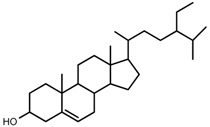	m.p., TLC
**9**	daucosterol	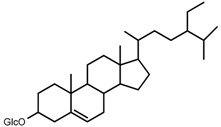	m.p., TLC

^1^H-NMR, ^1^H-nuclear magnetic resonance spectroscopy; ^13^C-NMR, ^13^C-nuclear magnetic resonance spectroscopy; HMBC, heteronuclear multiple-bond correlation; HSQC, heteronuclear singular quantum correlation; m.p., melting point; UV, ultraviolet spectrum; CD, circular dichroism spectrum; HR-ESI-TOF-MS, high-resolusion electrospray-ionization time-of-flight mass spectrometry; TLC, thin-layer chromatography.

**Table 3:** The retention time (Rt) and mass (MS) characteristics of the main detected peaks in extracts of *Rhodiola rosea* L.

Compound No.	Compound Name	Molecular Formula	Molecular Weight	Rt (min)	[M + H]^+^/[M + Na]^+^	Error (ppm)
**1**	3,4,5-trihydroxybenzoic acid (2*S*-*cis*)-3,4-dihydro-5,7-dihydroxy-2-(3,4,5-trihydroxyphenyl)-2*H*-benzo[*b*]pyran-3-yl ester	C_22_H_18_O_11_	458.0849	5.10	459.0927	−0.7
**2**	herbacetin-3-*O*-β-d-glucopyranoside-7-*O*-α-l-rhamnoside	C_27_H_30_O_16_	610.1534	21.56	611.1612	0.8
**3**	kaempferol-7-*O*-α-l-rhamnoside	C_21_H_20_O_10_	432.1056	12.29	433.1135	0.7
**4**	Rhodiocyanoside A	C_11_H_17_NO_6_	450.2465	1.86	260.1138	1.2
**5**	Rosarin	C_20_H_28_O_10_	428.1682	9.76	451.1578	−0.4
**6**	Rosavin	C_20_H_28_O_10_	428.1682	10.58	451.1581	0.2
**7**	Rhodionin	C_21_H_20_O_11_	449.1084	21.60	449.1084	0.9
